# Association Between Physical Activity and Mental Health in Primary School Children

**DOI:** 10.3390/sports14070295

**Published:** 2026-07-10

**Authors:** Raúl Lendínez-Conejo, Agustín Aibar-Almazán, María del Carmen Carcelén-Fraile

**Affiliations:** 1 Faculty of Educational Sciences, University of Jaén, 23071 Jaen, Spain; rlc00032@red.ujaen.es; 2 Department of Health Sciences, Faculty of Health Sciences, University of Jaén, 35017 Jaen, Spain; 3 Department of Educational Sciences, Faculty of Social Sciences, University of Atlántico Medio, 35017 Las Palmas de Gran Canaria, Spain; 4 International Scientific Association on Innovation in Education and Health (ACIINES), 23007 Jaen, Spain; 5 International Network of Educational Law, 35017 Las Palmas de Gran Canaria, Spain

**Keywords:** physical activity, psychological well-being, anxiety, stress, childhood, primary education

## Abstract

Mental health during childhood is a major public health concern, and physical activity has been proposed as a modifiable behaviour associated with psychological well-being and emotional health. This study examined the associations between physical activity and mental health indicators, including psychological well-being, anxiety symptoms, and everyday stress, in primary school children. A cross-sectional study was conducted with 207 children from two schools in Jaén (Spain). Physical activity, psychological well-being, anxiety, and stress were assessed using validated questionnaires. Pearson correlations and multiple linear regression analyses adjusted for age, sex, and socioeconomic status were performed. Physical activity was positively associated with all dimensions of psychological well-being and with total psychological well-being (r = 0.182, *p* = 0.009). Significant inverse correlations were observed between physical activity and all anxiety dimensions, as well as total anxiety (r = −0.145, *p* = 0.037), although the association with total anxiety was not significant after adjustment (β = −0.149, *p* = 0.112). Physical activity was also negatively associated with all stress dimensions and remained significantly associated with lower total stress after adjustment (β = −0.253, *p* = 0.007). These findings indicate that higher levels of physical activity are associated with greater psychological well-being and lower everyday stress in primary school children. However, given the cross-sectional design, these associations should not be interpreted as causal relationships. Longitudinal studies are needed to clarify the directionality of these associations.

## 1. Introduction

Mental health during childhood has emerged as a major public health concern worldwide [1]. The World Health Organization estimates that approximately one in seven children’s and adolescent’s experiences mental health difficulties, with anxiety-related symptoms and emotional problems representing some of the most prevalent conditions during these developmental stages [2]. Poor mental health during childhood is associated with adverse academic, social, and health outcomes and substantially increases the risk of developing psychiatric disorders later in life [3,4]. Consequently, the identification of modifiable lifestyle factors that may promote psychological well-being and prevent emotional difficulties during childhood has become a priority for researchers, educators, and public health professionals [5].

Among these factors, physical activity has received considerable attention due to its broad benefits for physical and mental health [6]. It is currently considered one of the most important lifestyle behaviors for promoting health and preventing multiple physical and psychological illnesses throughout the lifespan [7,8]. Current international guidelines recommend that children accumulate at least 60 min of moderate-to-vigorous physical activity daily [9]. However, global surveillance data indicate that a substantial proportion of children do not meet these recommendations, underscoring the need for a better understanding of the consequences of insufficient physical activity on health outcomes [10].

The relationship between physical activity and mental health is supported by several theoretical frameworks and biological mechanisms [11]. From a neurobiological perspective, regular physical activity promotes neuroplasticity and increases the availability of neurotransmitters and neurotrophic factors, including serotonin, dopamine, endorphins, and brain-derived neurotrophic factor (BDNF), all of which have been linked to emotional regulation and psychological well-being [12]. Furthermore, physical activity may reduce chronic activation of the hypothalamic–pituitary–adrenal axis and lower cortisol concentrations, thereby contributing to a more adaptive stress response [13,14]. Beyond biological mechanisms, psychological theories suggest that participation in physical activity enhances self-efficacy, perceived competence, self-esteem, and autonomy [15]. Likewise, engagement in sport and active play provides opportunities for social interaction, peer support, and social connectedness, factors consistently associated with better mental health outcomes in youth [16].

Accumulating evidence suggests that physically active children tend to report higher levels of psychological well-being [17]. Systematic reviews and meta-analyses have demonstrated positive associations between physical activity and indicators such as life satisfaction, self-esteem, positive affect, and overall well-being [18,19]. In particular, regular participation in moderate-to-vigorous physical activity has been linked to greater psychological functioning and resilience during childhood and adolescence [20]. Nevertheless, much of the existing literature has focused on adolescents, whereas studies examining these relationships during late childhood remain comparatively scarce [21].

Similarly, physical activity has been proposed as a protective factor against stress [22,23]. Exposure to regular physical activity may improve stress resilience by facilitating adaptive physiological responses and promoting psychological resources that help children cope with daily challenges [24]. Previous studies have reported inverse associations between physical activity and stress in both adolescent populations [25]. Nevertheless, evidence among primary school children remains limited, despite the fact that stress-related experiences associated with academic demands, peer relationships, and family contexts begin to emerge during this developmental period [26,27].

Although substantial evidence supports associations between physical activity and mental health in adolescents and broader youth populations, fewer studies have focused specifically on children in late childhood. Children aged 9–11 years represent a developmental stage characterized by increasing autonomy, greater academic demands, and evolving peer relationships, all of which may influence both physical activity behaviors and mental health outcomes. Furthermore, previous research has often focused on single mental health indicators, whereas studies simultaneously examining psychological well-being, anxiety symptoms, and everyday stress in primary school-aged children remain limited. Therefore, further research is needed to better understand these associations during this important developmental period.

Therefore, the present study aimed to examine the associations between physical activity and multiple indicators of mental health, including psychological well-being, anxiety symptoms, and everyday stress, among primary school children aged 9–11 years. Based on previous evidence, it was hypothesized that higher levels of physical activity would be associated with greater psychological well-being and lower levels of anxiety and stress, even after adjusting for age, sex, and socioeconomic status.

## 2. Materials and Methods

### 2.1. Study Design

A cross-sectional observational study was conducted to analyze the association between physical activity levels and various mental health indicators, including psychological well-being, anxiety, and stress, in primary school children. Data collection was carried out at a single point in time using validated questionnaires administered to the participants.

The study was conducted in accordance with the ethical principles established in the Declaration of Helsinki for research involving human subjects. The research protocol was also approved by the Ethics Committee of the University of Jaén (code: 20260409/ABR.TFM). Prior to participation in the study, informed consent was obtained from the parents or legal guardians of all participants.

### 2.2. Participants

The sample consisted of 207 primary school students, aged between 9 and 11 years, from schools in the province of Jaén. Participants were selected using convenience sampling and participated voluntarily in the study after authorization from the schools and informed consent from their parents or legal guardians.

**Figure 1:**
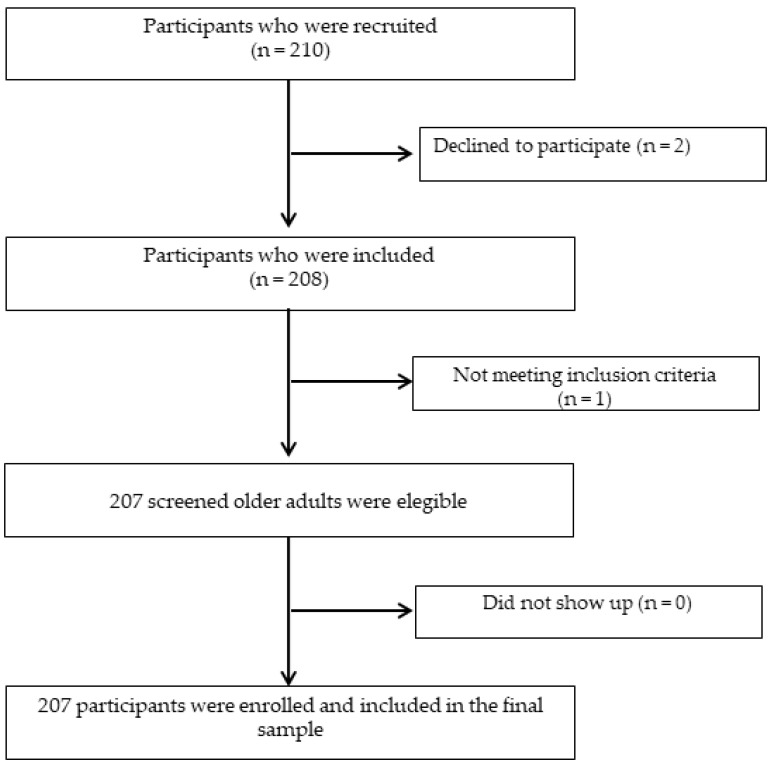
Participant recruitment and study flow.

A total of 210 primary school children were invited to participate in the study. Two students declined participation, resulting in 208 recruited participants. Subsequently, one participant was excluded due to incomplete questionnaire data. Therefore, the final analytical sample consisted of 207 children. Analyses were conducted using complete-case data, and no imputation procedures were required because all participants included in the final sample provided complete information for the variables analyzed (Figure 1).

The inclusion criteria were: (a) enrollment in Primary Education at one of the participating schools, (b) being between 9 and 11 years old, and (c) submitting informed consent signed by parents or legal guardians. The exclusion criteria were: (a) having cognitive or comprehension difficulties that prevented adequate completion of the questionnaires, (b) absence on the day of data collection, and (c) incomplete questionnaires or questionnaires with inconsistent data.

### 2.3. Outcomes

The variables analyzed were assessed using instruments previously validated for children and adolescents. All questionnaires were administered only once, yielding self-reported measures related to the different areas under study. The instruments were selected based on their adequate psychometric properties and their widespread use in the scientific literature.

#### 2.3.1. Physical Activity

Physical activity was assessed using the Physical Activity Questionnaire for Children (PAQ-C) [28], a self-administered instrument designed to evaluate the overall levels of physical activity performed by children and adolescents over the past seven days. The questionnaire consists of nine items that explore different contexts of physical activity, including sports, leisure-time exercise, school activity, and weekend activity. Responses are recorded using a 5-point Likert scale, with a final score calculated from the mean of the items, ranging from 1 to 5, where higher scores indicate greater levels of habitual physical activity. The PAQ-C has demonstrated adequate psychometric properties in children and adolescents, showing satisfactory levels of reliability and validity in different cultural and educational contexts. Physical activity was assessed using the Physical Activity Questionnaire for Children (PAQ-C), which provides a general measure of habitual physical activity performed during the previous seven days. Higher scores indicate higher levels of self-reported physical activity. In this study, the PAQ-C demonstrated good internal consistency (Cronbach’s alpha = 0.719).

#### 2.3.2. Psychological Well-Being

Psychological well-being was assessed using the Ryff Psychological Well-being Scales [29,30], an instrument designed to assess different dimensions of positive psychological functioning. The scale is composed of 39 items distributed in six dimensions: self-acceptance (items 1, 7, 13, 19, 25 and 31), mastery of the environmental (items 5, 11, 16, 22, 28 and 39), positive relationships (items 2, 8, 14, 20, 26 and 32), personal growth (items 24, 30, 34, 35, 36, 37 and 38), autonomy (items 3, 4, 9, 10, 15, 21, 27 and 33) and purpose in life (items 6, 12, 17, 18, 23 and 29). The responses are recorded using a Likert-type scale from 1 (totally disagree) to 6 (totally agree), obtaining a total psychological well-being score and specific scores for each dimension evaluated. The total score ranges between 39 and 234 points, where higher scores reflect higher levels of psychological well-being. Likewise, each dimension presents specific ranges of interpretation depending on the number of items that comprise it. The Ryff Scales have shown adequate psychometric properties and wide use in the adolescent and adult population. In this study, Ryff’s psychological well-being scales demonstrated good internal consistency (Cronbach’s alpha = 0.805).

#### 2.3.3. Anxiety Symptoms

Anxiety symptoms were assessed using the Screen for Child Anxiety Related Emotional Disorders (SCARED) [31,32], an instrument designed to identify anxiety symptoms in children and adolescents. This study used the self-reported version (SCARED-Child), which consists of 41 items distributed across five dimensions related to anxiety disorders described in the DSM: somatization/panic, generalized anxiety, separation anxiety, social phobia, and truancy. Responses were recorded using a 3-point Likert scale, yielding a total anxiety score and specific scores for each subscale, with higher scores reflecting a greater presence of anxiety symptoms. The SCARED has demonstrated adequate psychometric properties, as well as good levels of reliability and validity in children and adolescents. In this study, the SCARED showed good internal consistency (Cronbach’s alpha = 0.790).

#### 2.3.4. Stress

Children’s everyday stress was assessed using the Children’s Everyday Stress Inventory (IECI) [33], an instrument designed to evaluate the presence of common stressful situations in children. The questionnaire consists of 22 dichotomous (yes/no) items, in which participants indicate whether certain situations, demands, or problems have occurred during the past year. The IECI assesses three main dimensions: health and psychosomatic problems (8 items), school stress (7 items), and family stress (7 items). Raw scores for each subscale are obtained by counting affirmative responses to the corresponding items, and an overall everyday stress score is calculated by summing the scores of the three subscales. Higher scores indicate greater levels of everyday stress in each assessed area and greater overall stress. The instrument is designed for children between 6 and 12 years of age and has demonstrated adequate psychometric properties, as well as satisfactory levels of reliability and validity. In this study, the IECI demonstrated good internal consistency (Cronbach’s alpha = 0.829).

#### 2.3.5. Confounding Variables

Age was considered a potential confounding variable due to the developmental changes that occur during primary education, which can influence both physical activity levels and the psychological variables assessed. During this developmental stage, progressive changes occur in psychological well-being, emotional regulation, and anxiety symptoms, as well as variations in physical activity patterns. Previous research has shown that participation in physical activity and various mental health indicators differ according to developmental stage in children and adolescents [34].

Sex was also considered a potential confounding factor, given that previous studies have described differences between boys and girls in both physical activity levels and various mental health indicators. Specifically, boys tend to exhibit higher levels of moderate-to-vigorous physical activity, while girls show higher levels of anxiety symptoms and everyday stress [35].

Family socioeconomic status (SES) was also included as a confounding variable due to its potential influence on physical activity habits and children’s psychological well-being. Previous studies have indicated that a higher SES is associated with better access to sports resources, extracurricular activities, and healthy environments, as well as with better mental health indicators in children and adolescents [36]. Socioeconomic status was assessed using a sociodemographic question completed by parents/legal guardians. Parents/legal guardians classified their family socioeconomic status into one of three predefined categories: low, medium, or high.

### 2.4. Sample Size Calculation

An a priori sample size estimation was conducted using G*Power 3.1.9.7 for linear multiple regression, fixed model, R^2^ deviation from zero. Assuming a small-to-medium effect size (f^2^ = 0.06), α = 0.05, statistical power = 0.80, and four predictors, the minimum required sample size was 204 participants. Therefore, the final analytical sample of 207 children was considered sufficient for the planned regression analyses.

### 2.5. Procedure

First, the management teams of the participating schools were contacted to inform them about the objectives and characteristics of the study, and their authorization to carry out the research was subsequently requested. Once approval was obtained from the schools and the Ethics Committee of the University of Jaén. Written informed consent was obtained from parents or legal guardians prior to participation. In addition, all children provided age-appropriate assent before completing the study procedures.

Data collection took place during school hours at the educational centers, in a single assessment session. The questionnaires were administered collectively in the classroom under the supervision of the research team, ensuring the confidentiality and anonymity of the collected information at all times. Before beginning, participants were given instructions on how to complete the instruments, and any questions they had about the questionnaires were answered.

Participants completed instruments aimed at assessing children’s physical activity, psychological well-being, anxiety and daily stress, as well as a brief sociodemographic questionnaire. The estimated time to complete the battery of questionnaires was approximately 30–40 min.

### 2.6. Statistical Analysis

Statistical analyses were performed using IBM SPSS Statistics version 22.0 (IBM Corp., Armonk, NY, USA). Descriptive statistics are presented as means and standard deviations for continuous variables and frequencies and percentages for categorical variables. Pearson correlation analyses were conducted to examine bivariate associations between physical activity and mental health indicators. Multiple linear regression analyses were performed to investigate the associations between physical activity and psychological well-being, anxiety, and everyday stress after adjustment for age, sex, and socioeconomic status. Prior to regression analyses, the distributions of anxiety and everyday stress scores were visually inspected using histograms and normal probability plots. Although slight skewness was observed, no severe departures from normality were detected. Given the sample size (N = 207), ordinary least squares regression was considered appropriate and no transformation of the outcome variables was required. Sex was coded as 0 = boys and 1 = girls. Socioeconomic status was coded according to the predefined categories reported by parents/legal guardians. For each regression model, adjusted R^2^ values and 95% confidence intervals (95% CI) for regression coefficients were calculated. Additionally, the assumptions of ordinary least squares regression were evaluated. Linearity and homoscedasticity were assessed through visual inspection of scatterplots of standardized residuals against standardized predicted values. Normality of residuals was evaluated using histograms and normal probability (P–P) plots. Multicollinearity was assessed using tolerance and variance inflation factor (VIF) statistics, and influential observations were examined using Cook’s distance values. No substantial violations of regression assumptions were identified.

## 3. Results

### 3.1. Characteristics of the Participants

The study included 207 primary school children aged between 9 and 11 years (mean age = 9.98 ± 0.73 years), of whom 94 were boys (45.4%) and 113 were girls (54.6%). Regarding socioeconomic status, 28.0% of participants were classified as having a low/medium-low socioeconomic status, 42.5% as medium, and 29.5% as medium-high/high. The mean physical activity score was 3.08 ± 0.68 points, with similar values observed in boys and girls. No statistically significant sex differences were found for age, socioeconomic status, physical activity levels, psychological well-being dimensions, anxiety symptoms, or everyday stress indicators (all *p* > 0.05). Overall, participants reported moderate levels of psychological well-being (166.50 ± 34.08), anxiety (27.91 ± 15.93), and daily stress (7.67 ± 4.89). Detailed descriptive characteristics of the sample according to sex are presented in Table 1.

### 3.2. Correlations Between Physical Activity and Psychological Well-Being, Anxiety and Stress in Children

Significant associations were observed between levels of physical activity and different dimensions of psychological well-being, anxiety, and childhood stress (Table 2). Regarding psychological well-being, physical activity showed positive and significant correlations with self-acceptance (r = 0.214; *p* = 0.002), positive relationships (r = 0.240; *p* < 0.001), autonomy (r = 0.147; *p* = 0.034), environmental mastery (r = 0.164; *p* = 0.018), purpose in life (r = 0.195; *p* = 0.005), personal growth (r = 0.167; *p* = 0.016), and overall psychological well-being (r = 0.182; *p* = 0.009).

Regarding anxiety, physical activity showed significant negative associations with the dimensions of panic (r = −0.177; *p* = 0.011), generalized anxiety (r = −0.165; *p* = 0.017), separation anxiety (r = −0.180; *p* = 0.009), social anxiety (r = −0.190; *p* = 0.006), school anxiety (r = −0.200; *p* = 0.004), and total anxiety (r = −0.145; *p* = 0.037). These results indicate that higher levels of physical activity were associated with lower levels of anxiety symptoms in the studied sample.

Regarding children’s daily stress, physical activity showed significant negative associations with health-related stress (r = −0.177; *p* = 0.011), school stress (r = −0.141; *p* = 0.043), family stress (r = −0.169; *p* = 0.015), and total stress (r = −0.166; *p* = 0.017). These results indicate that higher levels of physical activity were associated with lower levels of everyday stress in the different areas assessed (Table 2).

### 3.3. Association Between Physical Activity and Psychological Well-Being

A multiple linear regression analysis was performed to examine the association between physical activity and overall psychological well-being, adjusting for age, sex, and family socioeconomic status. The overall model was statistically significant (F = 2.755; *p* = 0.029), explaining 5.2% of the variance in overall psychological well-being (R^2^ = 0.052; adjusted R^2^ = 0.033). Physical activity showed a positive and significant association with overall psychological well-being (β = 0.297; *p* = 0.001; 95% CI: 5.821 to 24.171), indicating that higher levels of physical activity were associated with higher levels of psychological well-being. Age showed a positive trend toward statistical significance (β = 0.174; *p* = 0.061; 95% CI: −0.373 to 16.706), while sex and socioeconomic status did not show significant associations with overall psychological well-being (*p* > 0.05) (Table 3).

### 3.4. Association Between Physical Activity and Total Anxiety

In addition, a multiple linear regression analysis was performed to examine the association between physical activity and total anxiety, adjusting for age, sex, and family socioeconomic status. The overall model was not statistically significant (F = 1.790; *p* = 0.132), explaining 3.4% of the variance in total anxiety (R^2^ = 0.034). Physical activity showed a negative association with total anxiety (β = −0.149; *p* = 0.112), indicating a trend toward lower anxiety levels in participants with higher levels of physical activity, although the association did not reach statistical significance. Age, sex, and socioeconomic status did not show significant associations with total anxiety (*p* > 0.05) (Table 4).

### 3.5. Association Between Physical Activity and Total Stress

A multiple linear regression analysis was performed to examine the association between physical activity and total stress, adjusting for age, sex, and family socioeconomic status. The overall model approached statistical significance (F = 2.400; *p* = 0.051), explaining 4.5% of the variance in total stress (R^2^ = 0.045; adjusted R^2^ = 0.026). Physical activity was significantly and negatively associated with total stress (β = −0.253; *p* = 0.007), indicating that higher levels of physical activity were associated with lower levels of stress. Age, sex, and socioeconomic status were not significantly associated with total stress (*p* > 0.05) (Table 5).

## 4. Discussion

The present study examined the association between physical activity and several indicators of mental health, including psychological well-being, anxiety symptoms, and everyday stress, in primary school children aged 9–11 years. Overall, the findings partially supported the initial hypothesis. Higher levels of physical activity were positively associated with psychological well-being and negatively associated with everyday stress, even after adjusting for age, sex, and family socioeconomic status. In contrast, although physical activity showed significant inverse correlations with all anxiety dimensions, its association with total anxiety did not remain statistically significant in the adjusted regression model. These results suggest that physical activity may be more consistently related to positive psychological functioning and stress regulation than to global anxiety symptoms in this sample.

The strongest finding of the present study was the positive association between physical activity and psychological well-being. Children reporting higher levels of physical activity showed higher scores in overall psychological well-being and in all its dimensions, including self-acceptance, positive relationships, autonomy, environmental mastery, purpose in life, and personal growth. This result is consistent with the growing body of evidence indicating that physical activity is not only associated with the absence of psychological symptoms, but also with positive indicators of mental health. Previous systematic reviews have consistently reported that physically active children and adolescents tend to exhibit greater psychological well-being, higher self-esteem, and better emotional functioning than their less active peers [37,38]. Similarly, umbrella reviews have highlighted that regular participation in physical activity is positively associated with subjective well-being, life satisfaction, and overall mental health across childhood and adolescence [39]. These findings are further supported by evidence suggesting that physical activity is associated with psychological well-being and with factors such as everyday competence, social connectedness, and self-regulation processes [40]. These findings are especially relevant because psychological well-being represents a positive and multidimensional construct, rather than merely the absence of mental illness. From this perspective, the results of the present study suggest that active children may perceive themselves as more competent, more autonomous, better able to manage their environment, and more connected with others. This interpretation is aligned with theoretical models proposing that physical activity has been associated with well-being through the satisfaction of basic psychological needs, including competence, autonomy, and relatedness. Participation in physical activity and sport provides children with opportunities to experience mastery, set goals, cooperate with peers, receive social support, and develop a sense of belonging, all of which may strengthen positive psychological functioning.

The positive association observed between physical activity and the dimension of positive relationships deserves particular attention. In childhood, peer interactions are a central component of socioemotional development, and active play, physical education, and sport may provide structured and unstructured contexts in which children interact, cooperate, negotiate rules, and build friendships. Previous research has shown that peer relationships are closely linked to well-being and academic adjustment during childhood [41,42]. Therefore, the social context in which physical activity occurs may partly explain why more active children in the present study showed better scores in positive relationships and overall well-being. This is important because it suggests that the associations between physical activity and mental health may depend not only on movement itself, but also on the quality of the social experiences associated with it [43].

A second relevant finding was the inverse association between physical activity and everyday stress. Physical activity was negatively correlated with health-related, school-related, family-related, and total stress. Moreover, the association between physical activity and total stress remained statistically significant after adjustment for age, sex, and socioeconomic status. This finding is consistent with previous studies suggesting that physically active children and adolescents tend to report lower levels of stress. For example, Kim et al. [25] reported associations between physical activity type and frequency and everyday stress among adolescents, while Martín-Rodríguez and González-Prieto [22] concluded in a systematic review that physical activity is generally associated with lower everyday stress and better mental health. The present study extends this evidence to primary school children, a population in which stress has been less frequently studied despite the emergence of school, family, and peer-related demands during this developmental stage.

Several mechanisms have been proposed in the literature to explain the observed associations between physical activity and lower stress. At the biological level, previous research has reported associations between regular physical activity, physiological regulation of stress-related systems and enhanced resilience to environmental challenges. Evidence suggests that exercise may promote neuroplasticity and brain resilience through changes in neurotrophic factors, neurotransmitter availability, and other biological pathways involved in stress adaptation [24]. Furthermore, physical activity has been proposed to influence mental health through multiple neurobiological, psychosocial, and behavioural mechanisms, including improvements in self-regulation and adaptive coping processes [44]. These adaptations may help children cope more effectively with daily stressors, including academic pressure, interpersonal difficulties, and family-related demands.

From a psychological perspective, physical activity has been associated with lower everyday stress by improving self-regulation, emotional control, coping skills, and resilience. This interpretation is supported by recent reviews showing that physical activity is positively associated with psychological resilience in young people. Resilience may be particularly relevant in school-aged children, as it allows them to adapt to challenges and recover from stressful experiences. Therefore, the negative association between physical activity and stress observed in this study could reflect both physiological regulation and the development of psychological resources that help children manage everyday demands.

Regarding anxiety, the present study found significant negative correlations between physical activity and all anxiety dimensions, including panic, generalized anxiety, separation anxiety, social anxiety, school anxiety, and total anxiety. These findings are in line with previous evidence suggesting that physical activity may be associated with lower anxiety symptoms in children and adolescents. Recent umbrella reviews and meta-analyses have concluded that exercise can reduce anxiety symptoms in young populations, although effect sizes and certainty of evidence vary across studies. For example, Ahn and Fedewa [45] reported that physically active children tend to exhibit fewer emotional and anxiety-related problems than their less active peers. Likewise, Rebar et al. [46] concluded that regular physical activity exerts association with lower anxiety symptoms, supporting the role of exercise as a factor associated with mental health, but with adults.

However, in the present study, the association between physical activity and total anxiety did not remain significant in the adjusted regression model. This finding should not necessarily be interpreted as evidence of the absence of a relationship, but rather as an indication that the association between physical activity and anxiety may be weaker and more complex than those observed for psychological well-being and stress. Anxiety symptoms in children are influenced by multiple determinants, including temperament, family environment, academic pressure, peer relationships, sleep quality, adverse life experiences, and genetic vulnerability. Furthermore, anxiety is a heterogeneous construct comprising different symptom domains that may not be equally sensitive to physical activity. In the present study, significant associations were observed for specific dimensions such as social anxiety and school anxiety, which may be particularly influenced by contexts involving peer interaction, perceived competence, and participation in school-based activities. However, when all anxiety dimensions were combined into a global score, these specific associations may have been diluted. Consequently, future research should examine whether physical activity is differentially associated with specific anxiety profiles rather than with overall anxiety symptoms.

The developmental stage of the participants may also help explain the findings. Children aged 9–11 years are in a transitional period between middle childhood and early adolescence, characterized by increasing autonomy, growing academic demands, and greater importance of peer relationships. At this age, psychological well-being and stress may be more immediately influenced by daily routines and school experiences, whereas clinically relevant anxiety symptoms may depend more strongly on stable individual and contextual risk factors. This could explain why physical activity showed robust associations with well-being and stress, but a weaker adjusted association with total anxiety.

Although physical activity was significantly associated with psychological well-being and stress, the proportion of variance explained by the regression models was relatively modest. Similarly, the observed correlation coefficients were generally small in magnitude, indicating modest associations between physical activity and the mental health indicators examined. This finding is unsurprising given the multifactorial nature of mental health during childhood, which is shaped by a complex interaction of biological, psychological, family, school, and social influences. Nevertheless, even modest associations may be meaningful from a public health perspective when the exposure of interest is modifiable, accessible, and scalable. Therefore, the observed relationships should not be interpreted as strong effects, but rather as potentially relevant associations within a broader network of factors influencing children’s mental health.

The present findings have several practical implications. First, they highlight the relevance of physical activity as a behaviour associated with psychological well-being and everyday stress during primary school years. Schools represent particularly important settings for promoting physical activity because they provide access to most children and may help reduce inequalities in opportunities for participation. However, given the cross-sectional design of the present study, these findings should not be interpreted as evidence that increasing physical activity will necessarily improve mental health outcomes. Several strengths should be acknowledged. The study focused on children aged 9–11 years, an age group that has received less attention than adolescents in the physical activity and mental health literature. In addition, multiple dimensions of mental health were assessed simultaneously, including psychological well-being, anxiety symptoms, and everyday stress. The use of validated instruments and adjustment for relevant sociodemographic factors further strengthens the reliability of the findings.

However, some limitations must also be considered. The cross-sectional design involved the assessment of all variables at a single time point, precluding causal inference. In addition, all variables were assessed using self-report measures, which may be subject to recall and social desirability bias. Furthermore, physical activity was assessed using the Physical Activity Questionnaire for Children (PAQ-C) rather than objective methods such as accelerometry. Although the PAQ-C is a widely used and validated instrument, self-reported physical activity may be affected by reporting inaccuracies and may not reflect actual activity levels with the same precision as objective measures. Furthermore, the convenience sample recruited from two schools in the province of Jaén may limit the generalizability of the findings. Although analyses were adjusted for age, sex, and socioeconomic status, other potentially relevant factors, including sleep quality, screen time, family functioning, academic performance, and previous mental health history, were not considered. In addition, biological maturation was not assessed. Although chronological age was included as a covariate, indicators of biological maturation (e.g., bone age or maturity-related anthropometric measures) may provide a more accurate representation of developmental status and should be considered in future research. Although the regression models were adjusted for age, sex, and socioeconomic status, other factors that may influence both physical activity and mental health outcomes were not assessed. These include sleep characteristics, sedentary behavior, body mass index, family functioning, parental mental health, peer relationships, bullying, academic demands, chronic health conditions, previous mental health diagnoses, and participation in organized sports. Consequently, residual confounding cannot be ruled out, and the observed associations should be interpreted with caution.

Future studies should employ longitudinal and intervention-based designs to clarify the directionality of the observed associations and identify the characteristics of physical activity programmes that are most beneficial for children’s mental health. Further research should also explore potential mediating mechanisms, such as resilience, self-esteem, social support, emotional regulation, and sleep, as well as incorporate objective measures of physical activity to improve assessment precision.

## 5. Conclusions

The findings of the present study suggest that higher levels of physical activity are associated with better mental health outcomes among primary school children. Specifically, physical activity was positively associated with overall psychological well-being and negatively associated with everyday stress, even after adjusting for age, sex, and socioeconomic status. Although significant inverse correlations were observed between physical activity and anxiety symptoms, the association with total anxiety did not remain significant in the adjusted regression model.

These results highlight the existence of significant associations between physical activity and several indicators of mental health, particularly psychological well-being and everyday stress. Given the cross-sectional nature of the study, causal relationships cannot be established. Therefore, future longitudinal and intervention-based research is needed to clarify the directionality and underlying mechanisms of these associations. The findings support the relevance of considering physical activity as a factor associated with mental health during childhood and underscore the need for further research in this area.

## Figures and Tables

**Table 1:** Descriptive characteristics of the sample according to sex.

Outcomes		Total (N = 207)	Boys (N = 94)	Girls (N = 113)	*p*
AGE (YEARS)		9.98 ± 0.73	10.02 ± 0.70	9.94 ± 0.75	0.414
ECONOMIC STATUS	Low/low-medium	58 (28.00)	30 (31.90)	28 (24.80)	0.850
	Medium	88 (42.50)	39 (41.50)	49 (43.40)	
	Medium-high/high	61 (29.50)	25 (26.60)	36 (31.90)	
PHYSICAL ACTIVITY		3.08 ± 0.68	3.06 ± 0.64	3.08 ± 0.6471	0.823
PSYCHOLOGICAL WELL-BEING	Self-acceptance	26.78 ± 6.37	26.83 ± 6.03	26.73 ± 6.66	0.915
	Positive relationships	26.39 ± 6.32	26.39 ± 5.93	26.38 ± 6.66	0.988
	Autonomy	40.64 ± 6.93	40.24 ± 6.39	40.97 ± 7.36	0.453
	Environmental mastery	20.67 ± 4.51	20.51 ± 4.20	20.80 ± 4.76	0.651
	Purpose in life	27.02 ± 4.99	27.00 ± 4.70	27.04 ± 5.24	0.960
	Personal growth	24.85 ± 5.58	24.79 ± 5.20	24.85 ± 5.58	0.892
	Total psychological well-being	166.50 ± 34.08	166.50 ± 33.88	166.53 ± 35.94	0.988
ANXIETY	Panic	7.15 ± 5.66	7.17 ± 5.47	7.14 ± 5.84	0.971
	Generalized anxiety	7.70 ± 4.50	7.71 ± 4.31	7.68 ± 4.68	0.960
	Separation anxiety	5.92 ± 3.59	5.93 ± 3.38	5.92 ± 3.76	0.992
	Social anxiety	5.60 ± 3.39	5.69 ± 3.33	5.53 ± 3.45	0.735
	School anxiety	1.91 ± 1.67	1.93 ± 1.68	1.89 ± 1.67	0.856
	Total anxiety	27.91 ± 15.93	28.09 ± 15.38	27.76 ± 16.44	0.885
EVERYDAY STRESS IN CHILDREN	Health-related stress	2.84 ± 1.86	2.81 ± 1.79	2.87 ± 1.93	0.822
	School-related stress	2.61 ± 1.68	2.62 ± 1.59	2.61 ± 1.76	0.978
	Family stress	2.21 ± 1.45	2.18 ± 1.34	2.24 ± 1.54	0.742
	Total stress	7.67 ± 4.89	7.61 ± 4.60	7.73 ± 4.89	0.862

Note. Data are presented as mean ± standard deviation or absolute frequency (percentage). IPAQ-C = Physical Activity Questionnaire for Children; SCARED = Screen for Child Anxiety Related Emotional Disorders.

**Table 2:** Correlations between physical activity and psychological well-being, anxiety, and stress indicators.

Outcomes		Level of Physical Activity (r)	*p*
Psychological well-being	Self-acceptance	0.214	0.002
	Positive relationships	0.240	<0.001
	Autonomy	0.147	0.034
	Environmental mastery	0.164	0.018
	Purpose in life	0.195	0.005
	personal growth	0.167	0.016
	Total psychological well-being	0.182	0.009
Anxiety	Panic	−0.177	0.011
	Generalized anxiety	−0.165	0.017
	Separation anxiety	−0.180	0.009
	Social anxiety	−0.190	0.006
	School anxiety	−0.200	0.004
	Total anxiety	−0.145	0.037
Everyday Stress in Children	Health-related stress	−0.177	0.011
	School-related stress	−0.141	0.043
	Family stress	−0.169	0.015
	Total stress	−0.166	0.017

Note. r = Pearson correlation coefficient. All statistically significant correlations remained significant after application of the Benjamini–Hochberg false discovery rate (FDR) correction.

**Table 3:** Association between physical activity and total psychological well-being using multiple linear regression.

Predictor Variables	B	SE	β	t	*p*	95% CI Lower	95% CI Upper
Physical activity (PAQ-C)	14.996	4.653	0.297	3.223	0.001	5.821	24.171
Age	8.166	4.331	0.174	1.886	0.061	−0.373	16.706
Sex	0.680	4.704	0.010	0.144	0.885	−8.596	9.955
Socioeconomic level	−1.966	3.084	−0.044	−0.637	0.525	−8.047	4.116

Note: N = 207. Adjusted R^2^ = 0.033. B = unstandardized coefficient; SE = standard error; β = standardized coefficient; CI = confidence interval.

**Table 4:** Association between physical activity and total anxiety using multiple linear regression.

Predictor Variables	B	SE	β	t	*p*	95% CI Lower	95% CI Upper
Physical activity (PAQ-C)	−3.505	2.195	−0.149	−1.596	0.112	−7.834	0.824
Age	−0.241	2.043	−0.011	−0.118	0.906	−4.271	3.788
Sex	−0.569	2.219	−0.018	−0.256	0.798	−4.945	3.807
Socioeconomic level	2.412	1.455	0.115	1.657	0.099	−0.458	5.281

Note: N = 207. Adjusted R^2^ = 0.015. B = unstandardized coefficient; SE = standard error; β = standardized coefficient.

**Table 5:** Association between physical activity and total stress using multiple linear regression.

Predictor Variables	B	SE	β	t	*p*	95% CI Lower	95% CI Upper
Physical activity (PAQ-C)	−1.828	0.670	−0.253	−2.729	0.007	−3.149	−0.507
Age	−0.896	0.623	−0.133	−1.438	0.152	−2.126	0.333
Sex	0.012	0.677	0.001	0.018	0.986	−1.323	1.347
Socioeconomic level	0.574	0.444	0.089	1.293	0.197	−0.301	1.449

Note: N = 207. Adjusted R^2^ = 0.026. B = unstandardized coefficient; SE = standard error; β = standardized coefficient.

## Data Availability

The data presented in this study are not publicly available due to privacy and ethical restrictions related to the participation of minors. Data may be available from the corresponding author upon reasonable request.

## Abbreviations

The following abbreviations are used in this manuscript:

PAQ-C	Physical Activity Questionnaire for Children
SCARED	Screen for Child Anxiety Related Emotional Disorders
IECI	Children’s Everyday Stress Inventory
SES	Socioeconomic Status
DSM	Diagnostic and Statistical Manual of Mental Disorders
BDNF	Brain-Derived Neurotrophic Factor
HPA	Hypothalamic–Pituitary–Adrenal
WHO	World Health Organization
